# Malignant Pleural Effusions—A Window Into Local Anti-Tumor T Cell Immunity?

**DOI:** 10.3389/fonc.2021.672747

**Published:** 2021-04-27

**Authors:** Nicola Principe, Joel Kidman, Richard A. Lake, Willem Joost Lesterhuis, Anna K. Nowak, Alison M. McDonnell, Jonathan Chee

**Affiliations:** ^1^ National Centre for Asbestos Related Diseases, Institute for Respiratory Health, University of Western Australia, Nedlands, WA, Australia; ^2^ School of Biomedical Sciences, University of Western Australia, Crawley, WA, Australia; ^3^ Telethon Kids Institute, Perth, WA, Australia; ^4^ School of Medicine, University of Western Australia, Crawley, WA, Australia

**Keywords:** malignant pleural effusions (MPE), T cells, immune checkpoint therapy, checkpoint receptors, memory T cells

## Abstract

The success of immunotherapy that targets inhibitory T cell receptors for the treatment of multiple cancers has seen the anti-tumor immune response re-emerge as a promising biomarker of response to therapy. Longitudinal characterization of T cells in the tumor microenvironment (TME) helps us understand how to promote effective anti-tumor immunity. However, serial analyses at the tumor site are rarely feasible in clinical practice. Malignant pleural effusions (MPE) associated with thoracic cancers are an abnormal accumulation of fluid in the pleural space that is routinely drained for patient symptom control. This fluid contains tumor cells and immune cells, including lymphocytes, macrophages and dendritic cells, providing a window into the local tumor microenvironment. Recurrent MPE is common, and provides an opportunity for longitudinal analysis of the tumor site in a clinical setting. Here, we review the phenotype of MPE-derived T cells, comparing them to tumor and blood T cells. We discuss the benefits and limitations of their use as potential dynamic biomarkers of response to therapy.

## Malignant Pleural Effusion Is a Complication in Thoracic Cancers

A malignant pleural effusion (MPE) is an abnormal accumulation of fluid in the pleural space associated with advanced stage disease and poor clinical outcomes (1, 2). These effusions are present at diagnosis in over 90% of patients with mesothelioma (3) and 40% of patients with advanced lung cancer (1), and are a common feature of metastatic disease to the lung in patients with breast cancer, lymphoma, ovarian and stomach cancers (2, 4). MPEs are an exudative fluid, resulting from increased vascular permeability, inflammation and plasma leakage caused in part by tumor cells blocking the outflow of fluid from the pleural space (5). This build up of fluid leads to symptoms of various severity including breathlessness, chest pain and cough (5), with current therapy consisting largely of palliative measures designed to drain or eliminate the pleural space to prevent accumulation of fluid (6–9). With the exception of pleurectomy, recurrent MPE can occur throughout disease progression.

As MPE is adjacent to both primary and metastatic lung tumor tissue, it is a unique peri-tumoral environment populated with tumor cells, cytokines, growth factors, enzymes and immune cells (10, 11). MPEs are routinely drained, providing an attractive option to longitudinally study the tumor microenvironment (TME) in thoracic cancers such as mesothelioma, where a major hurdle is the inability to collect serial tumor biopsies. Our review focuses on the adaptive immune cells in MPEs, and how they could inform responses to cancer immunotherapies.

## There Is an Urgent Need to Develop Biomarkers of Response to Immune Checkpoint Blockade

Immune checkpoint blockade (ICB) targeting T cell inhibitory receptors: cytotoxic T lymphocyte associated protein-4 (CTLA-4) and programmed cell death protein-1/ligand-1 (PD-1/PD-L1) have revolutionized cancer treatment. Single or dual agent ICB provides an durable survival benefit in patients with mesothelioma and non-small cell (NSCLC) lung cancer patients (12–15). Four ICB therapies: pembrolizumab, nivolumab, atezolizumab and durvulamab that target the PD-1/PD-L1 pathway are approved first and second-line treatments for patients with advanced NSCLC (16). Combination of ipilimumab (anti-CTLA-4) and nivolumab (anti-PD-1) provides a survival benefit over chemotherapy in malignant pleural mesothelioma (14). Platinum-based chemotherapies may synergize with ICB, with single arm studies showing that combination chemo-immunotherapy reduces tumor burden and shows promising progression-free and overall survival outcomes for mesothelioma (17–19). In addition, complete tumor regression has been observed in some NSCLC (20–22) and SCLC (23–25) patients treated with chemo-immunotherapy. Atezolizumab and durvalumab are also approved in combination with platinum-based chemotherapy for advanced SCLC patients (26). However, these best-case responses are only observed in a minority of patients. ICB is also expensive and can cause severe immune-related toxicities, highlighting the need to develop biomarkers that can accurately predict patient outcomes and inform clinical decisions (27). To date, several predictive biomarkers have been associated with ICB outcomes in some cancers, including intratumoral expression of the inhibitory receptor PD-L1 (28), the tumor mutational landscape (29), immune gene signatures within the TME (30, 31), and the presence of tumor infiltrating lymphocytes (TILs) and their expression of PD-1/PD-L1 (32–35). However, there is no common biomarker that can accurately predict ICB outcomes across different thoracic cancers, and there is a need to develop more nuanced biomarkers of response.

As ICB primarily acts through T cells, in-depth characterization of T cell subsets within the TME, and how they correlate with ICB outcomes, has been extensively investigated. CD8^+^ T cell subsets characterized by expression of activation/memory associated markers and their T cell receptor (TCR) usage have been linked with outcome to ICB (34, 36–39), highlighting the potential utility of T cell subsets to inform ICB responses. As T cells are also enriched in MPEs, they could offer insight into anti-tumor responses if they accurately reflect TIL phenotype, frequency and function. Longitudinal analysis of MPEs could reveal dynamic changes in the TME without the need for serial biopsies, and aid development of a biomarker of response.

Below, we review studies that have characterized matched MPE, TME and peripheral blood derived T cells, focusing on whether MPE T cells are similar in phenotype, function and specificity to their tumor counterparts. We also review changes in T cells derived from MPEs of patients undergoing ICB, and whether these changes were associated with treatment outcomes. Lastly, we discuss the unique opportunities and challenges a longitudinal study of MPE T cells brings, in terms of improving our understanding of therapeutic mechanisms, and developing a biomarker of response to ICB.

## Cellular Characteristics of Malignant Pleural Effusions Without ICB

MPEs contain multiple cell types including tumor cells, pleural mesothelial cells, and innate and adaptive immune cells (40). Innate immune cells in the MPE include monocytes, macrophages, neutrophils, mast cells, dendritic cells and natural killer cells (41). These cells release cytokines, growth factors and chemokines including monocyte chemotactic protein (MCP-1), vascular endothelial growth factor (VEGF), IL-8, IL-6, IL-1β, interferon gamma (IFN-*γ*), tumor necrosis factor alpha (TNF*α*), and transforming growth factor beta (TGFβ) (40, 42–48). These cytokines can be proinflammatory and in some cases protumorigenic, promoting angiogenesis, vascular permeability and protecting cancer cells from apoptosis. MPE have increased lactate dehydrogenase, and a lower pH than non-malignant pleural fluid, suggestive of an immunosuppressive environment (11, 41, 49). The characterization of MPE proteins, cytokine milieu, innate cells, tumor cells, and their relation to overall survival have been extensively reviewed elsewhere (50–52), so this review will focus on T cells.

### MPE Are Enriched With T Cells

The proportion of total T cells in the MPE is greater than in matched peripheral blood samples from both mesothelioma and lung cancer patients (53, 54). CD4^+^ T cells are the predominant T cell subset in MPE both prior to and after chemotherapy (41, 54–57). Of these CD4^+^ T cells, an increased proportion of regulatory T cells (CD4^+^CD25^+^) are recruited in MPEs by chemokines and pro-inflammatory cytokines, compared to matched peripheral blood (58–61). Despite this abundance of CD4^+^ T cells, several studies have shown that the CD4^+^/CD8^+^ T cell ratio in MPEs is similar to matched peripheral blood samples in patients with mesothelioma (54, 62). For lung cancer patients, CD8^+^ T cell frequencies were greater in the peripheral blood compared to the MPE for one study (53) but were similar between both compartments in others (41, 63), suggesting CD8^+^ T cell infiltration in the pleural space may be more cancer or chemotherapy specific.

Several recent studies have examined the effect of chemotherapy on the immune milieu of matched MPE and tumor samples. At baseline, MPEs contain a lower frequency of CD4^+^ and CD4^+^ regulatory (Foxp3^+^) T cells compared to matched NSCLC tumor tissue, whereas CD8^+^ T cells were increased in the MPE compared to tumor samples (63). After chemotherapy, matched MPEs and tumor tissue from patients with mesothelioma displayed similar proportions of CD3^+^ T cells, CD4^+^ helper (CD25^-^) and CD4^+^ regulatory (CD25^+^CD127^lo^) T cells post-chemotherapy (64), but similarly, CD8^+^ T cells were greater in the MPE than matched tumor tissue (10, 64). Increased pre- and post-chemotherapy frequencies of CD4^+^ T cells in MPE and tumors were associated with complete response and improved survival in chemotherapy treated mesothelioma patients (56, 65, 66). Post-chemotherapy regulatory T cell frequencies in tumors negatively associated with survival, but this association was not observed in matched MPE samples (64). Comparison of T cell proportions between tumor and MPE in these studies are limited by small sample sizes, and whether proportions of CD4^+^ and CD8^+^ T cells are similar in matched tumor and MPE samples are unknown.

MPEs are typically enriched with CD4^+^ T cells, particularly regulatory CD4^+^ T cells. CD4^+^/CD8^+^ T cell ratios in MPEs vary between patients, likely because of patient heterogeneity such as prior treatment, disease stage and amount of fluid drained. The surface phenotype, effector function, and differentiation status of MPE T cells offers further insight into the immune status of the MPE.

### MPE-Derived T Cells Express Inhibitory Checkpoint Receptors

T cells upregulate inhibitory checkpoint receptors in the presence of chronic tumor antigen exposure. Checkpoint receptor signaling inhibits T cell function, and immunosuppressive TMEs exploit these signaling pathways to curtail an effective anti-tumor response. Although CTLA-4 and PD-1 are the most common targets in ICB therapy, other inhibitory checkpoint receptors are expressed on TILs including TIM-3, LAG-3, TIGIT and PD-L1. Increased frequencies of CD8^+^PD-1^+^ T cells in tumors post anti-PD-1 treatment have been associated with complete and partial responses in NSCLC (34). Hence, the expression of checkpoint receptors on MPE T cells is of great interest because these T cells could be potential targets for ICB, and predictors of response.

The expression of inhibitory receptors on CD4^+^ and CD8^+^ T cells in MPE have been reported in multiple studies for mesothelioma and lung cancer. While this varies between patients, ~30% of CD4^+^ and ~40% of CD8^+^ T cells express PD-1 in the MPE (56, 62, 63) and these frequencies are greater than in matched peripheral blood T cells (62, 63, 67–70). Inhibitory receptors TIM-3, LAG-3, CTLA-4 and PD-L1 are also expressed on MPE CD4^+^ and CD8^+^ T cells at greater proportions than matched peripheral blood samples in mesothelioma and lung cancer patients (56, 62, 63, 67, 69). In addition, regulatory T cells constitutively express the inhibitory receptor TIGIT (71), and display increased expression of CTLA-4 and PD-1 in the MPE compared to peripheral blood (58, 63).

In comparison to tumor tissue, the frequencies of CD8^+^ T cells expressing PD-1 and TIM-3 are greater than the MPE prior to treatment (63). However, they are similar in frequency between the two compartments post-chemotherapy (64). For CD4^+^ helper (Foxp3^-^) T cells and regulatory (Foxp3^+^) T cells, the expression of PD-1 and TIM-3 are similar between matched MPE and tumor tissue both pre- and post-chemotherapy (63, 64). In addition, the proportion of CD4^+^LAG-3^+^ and CD8^+^LAG-3^+^ T cells in MPE after chemotherapy are similar to tumor tissue in one study (64), but not another (56). Co-expression of inhibitory receptors on T cells, in particular PD-1 and TIM-3, indicates further T cell dysfunction which has been reported to be unfavorable for ICB efficacy (37). To date, there is only one report of co-expression of these receptors, which found that the majority of CD4^+^PD-1^+^ and CD8^+^PD-1^+^ MPE T cells prior to treatment did not co-express LAG-3 or TIM-3. Less than 2% of CD8^+^ T cells were PD-1^+^TIM-3^+^ and less than 6% of CD4^+^ T cells were PD-1^+^LAG-3^+^ (68), suggesting that most of the CD8^+^ T cells in the MPE could be amenable to anti-PD-1 therapy.

MPE T cells are similar to TILs in that they both express increased inhibitory checkpoint receptors compared to blood T cells. Although the expression of inhibitory receptors TIM-3, PD-1 or LAG-3 on MPE T cells did not associate with improved survival post chemotherapy (64), inhibitory receptor co-expression on MPE T cells, and their correlation to ICB therapy outcomes are still of interest.

### MPE-Derived CD8^+^ T Cells Exhibit a Memory Phenotype

A hallmark of antigen-specific T cell responses is their ability to differentiate into memory T cells after activation, and mount a rapid response upon re-exposure to their cognate antigen. Memory CD8^+^ T cells are loosely classified into effector memory (T_EM_: CD45RO^+^/CD62L^-^, CD45RA^-^CCR7^-^), central memory (T_CM_: CD45RO^+^CD62L^+^, CD45RA^-^CCR7^+^) and resident memory (T_RM_: CD45RO^+^CD103^+^) subsets based on surface expression of differentiation markers and tissue localization. T_EM_ and T_CMs_ are generally found circulating in the peripheral blood and lymphatics, whilst T_RMs_ are non-circulatory and tissue tropic. Understanding CD8^+^ memory T cell differentiation status is crucial because inhibitory checkpoint receptors are highly expressed on memory CD8^+^ T cells in tumors (72–74) and are potential cellular targets of ICB. ICB also drives changes in CD8^+^ memory T cell differentiation (75). Importantly, tumor infiltration of memory T cell subsets and their gene signatures correlated with ICB response and overall survival in melanoma and lung cancer patients (36, 73). MPE-derived CD4^+^ and CD8^+^ T cells in mesothelioma and lung cancer exhibit a memory phenotype prior to treatment. MPEs have increased frequencies of T_CM_ and T_EM_ cells for both cancer types compared to peripheral blood (53, 57). MPEs also have greater frequencies of T_CM_ but reduced T_EM_ compared to non-malignant pleural fluid (54).

Memory T cell subsets found in MPE are phenotypically similar to subsets found in mesothelioma tumors but there are limited studies on matched samples. Both MPE and tumors have a greater frequency of T_EM_ cells than the circulation (76), suggesting that the proportion of T_EM_ cells in the MPE may reflect the TME. Recent studies have also shone a spotlight on the role of T_RMs_ in tumor immunosurveillance. Increased pre-treatment frequencies of T_RMs_ in tumors associate with improved survival (72, 74), and increase pre- or post-treatment frequencies associate with response to anti-PD-1 therapy in lung cancer patients (73). CD8^+^ T_RMs_ prior to chemotherapy have been reported in MPE of lung cancer patients, but in lesser proportions compared to matched tumor samples (77). Similar to their tumor counterparts, memory T cell subsets in the MPE could offer a predictor of therapeutic response.

### MPE-Derived CD8^+^ T Cells Have Impaired Effector Function

CD8^+^ T cells proliferate and produce effector molecules such as cytotoxic granules (granzyme B, perforin) and proinflammatory cytokines (IFN*γ*) to mediate tumor cell killing. The ability of T cells to produce effector molecules *ex vivo* is a measure of T cell effector function. Understanding the effector status of T cells is important as ICB induces activation and proliferation of circulating and intratumoral T cells which correlates with response (78, 79). While MPE CD8^+^ T cells can produce IFN*γ*, granzyme B and perforin *ex vivo*, the frequency of MPE T cells that secrete these molecules is reduced compared to T cells from matched peripheral blood samples (67, 70). Specifically, blood derived effector (CD45RA^+^CD27^-^) T cells have increased perforin secretion than these T cell subsets from the MPE (53). However, these reports have used non-specific stimuli *ex vivo* to measure effector molecule production from MPE and peripheral blood T cells. Antigen-specific assays are required to understand if impairment is restricted to tumor antigen-specific T cells only.

There are limited comparisons of matched MPE-derived T cell and TIL effector function. TILs and matched MPE T cells from advanced NSCLC patients were hypofunctional, with decreased frequency of CD8^+^IFN*γ* ^+^ T cells than tumors from patients with early stage NSCLC (80). Impaired effector function of MPE T cells could be due to an immunosuppressive environment characterized by high levels of TGFβ, tumor associated macrophages and myeloid derived suppressor cells (42, 55, 64, 67).

### Different CD4^+^ T Helper Cell Subsets Are Found in the MPE

Effector CD4^+^ T cells can differentiate into helper T cell (Th) subtypes which have been identified in MPE from people with lung cancer and mesothelioma. The CD4^+^ helper T cell subtypes include Th1, Th2, Th17, Th9 and Th22 which are each identified by unique transcriptional signatures, and production of different cytokines (81, 82). ICB induces expansion of effector Th1 and Th17 cells in the TME, therefore it is important to determine if Th subtypes in the MPE is associated with ICB outcomes (83–85).

Th1 cells are pro-inflammatory, secreting IFN*γ* to stimulate effector CD8^+^ T cell differentiation. Approximately 45% of CD4^+^ MPE T cells produce IFN*γ* indicating a predominant Th1 phenotype in the MPE which is greater in frequency than matched peripheral blood samples (53). In comparison to Th1, Th2 promotes humoral immunity by producing cytokines IL-4, IL-5 and IL-10. The balance of these two subsets in the MPE remains controversial. Some reports suggest the MPE favors the Th2 over the Th1 pathway in comparison to pleural fluid from tuberculosis patients (86, 87). However, IL-4 was detected in the MPE in some studies (86–88) but was below 1% or undetected in others (53, 55, 89). In addition, IL-4 was detected at low levels and IFN*γ* was undetected in both paired MPE and mesothelioma tumor supernatant in another study (55).

The role of Th17 cells in the TME also remains controversial. The production of IL-17 has been reported to stimulate recruitment of dendritic cells, NK cells and CD8^+^ T cells into the TME (90), but also can promote tumor growth through IL-17R signaling (91, 92). Frequencies of Th17 cells are greater in the MPE than peripheral blood and exhibit an T_EM_ phenotype (CD45RO^+^CD45RA) (93). The proportion of Th17 cells negatively correlated with regulatory T cells in the MPE, suggesting that regulatory T cells inhibited generation and differentiation of Th17 cells in the pleural space (61). For tumor tissue, one study found IL-17 in mesothelioma tumor supernatant but not in matched MPE (55).

In comparison, Th9 and Th22 cells suppress anti-tumor immunity. Both Th9 and Th22 cell proportions in the MPE are greater than the peripheral blood and also express an T_EM_ phenotype (CD45RO^+^CD45RA) in both compartments (94, 95). Th9 cells produce IL-9 which has been identified to promote tumor angiogenesis (96). Th9 cell frequencies correlate to regulatory T cell frequencies in the MPE, and higher Th9 cells in MPEs associated with poor survival in lung cancer patients (95). There are no reports of Th9 cells in matched MPE and TME. One study suggests that Th9 cells may infiltrate into the MPE from the circulation as CCR7 expression was decreased on Th9 cells in the MPE compared to matched blood (97). Th22 cells produce IL-22 which has been identified to promote migration and proliferation of cancer cells and resist apoptosis and chemotherapy (98). In NSCLC patients, IL-22 was greater in matched tumor tissue than MPE (99), and IL-22 expression in MPE promoted cancer cell migration (94), and protected cancer cells from apoptosis by chemotherapies (99).

Taken together, it is evident that multiple CD4^+^ T helper subtypes are present in the MPE, but varying frequencies of different subtypes have been reported. Further analysis is required to understand if any of these cell types in the MPE associate with ICB efficacy.

### MPE-Derived CD8^+^ T Cells Are Clonally Expanded, and Some Are Specific for Tumor Antigens

Importantly, tumor antigen-specific T cells can be found in the MPE. Co-culture of MPE-derived lymphocytes with tumor cells or known tumor antigens from lung cancer patients resulted in IFN*γ* production (41, 69) and CD137 expression (68), suggesting tumor reactivity (100). In addition, tumor reactive MPE-derived CD8^+^ T cells displayed a memory phenotype with checkpoint expression (PD-1^+^TIM-3^-^) (68). However, most studies have included a T cell expansion step prior to assessing tumor reactivity, so the actual proportion of MPE T cells specific for tumor-antigens is unclear.

In addition to screening for reactivity, T cell receptor (TCR) analyses are used to study antigen-specific T cell responses. Individual TCRα/β chains are highly variable across complementarity determining regions (CDR), the regions crucial for antigen-specificity. Antigen-specific clonal expansion can be estimated by quantifying the distribution of TCR variable genes, or CDR sequences. ICB induces a peripheral expansion of TCR clonotypes which correlates to clinical benefit in lung cancer (101, 102). In lung tumors, the TCR repertoire clonality and the number of expanded TCR clones was greater in ICB responders compared to non-responders post-treatment (38).

TCR analyses of matched MPE and blood from lung cancer patients revealed over expression of particular TCRβ variable genes in the MPE compared to matched blood samples (103), suggesting that MPE T cells had undergone clonal expansion. The presence of shared, highly expanded TCR clonotypes in MPE and tumors would greatly support the notion that T cells in both compartments are similar. High throughput TCR sequencing is used in this area, as shared TCRβs have been found in ascites and tumors in other studies (104). We previously reported that CD4^+^PD-1^+^ MPE T cells consist of distinct, clonally expanded TCRβs from CD4^+^PD-1^+^ T cells in matched peripheral blood  (62). TCR analyses of matched TILs and MPE T cells in thoracic cancers are currently limited, and would greatly inform the similarities in antigen-specificity between the compartments.

### MPE T Cells and TILs Exhibit Phenotypic Similarities, but the Extent of Similarity Is Unclear

**Figure 1:**
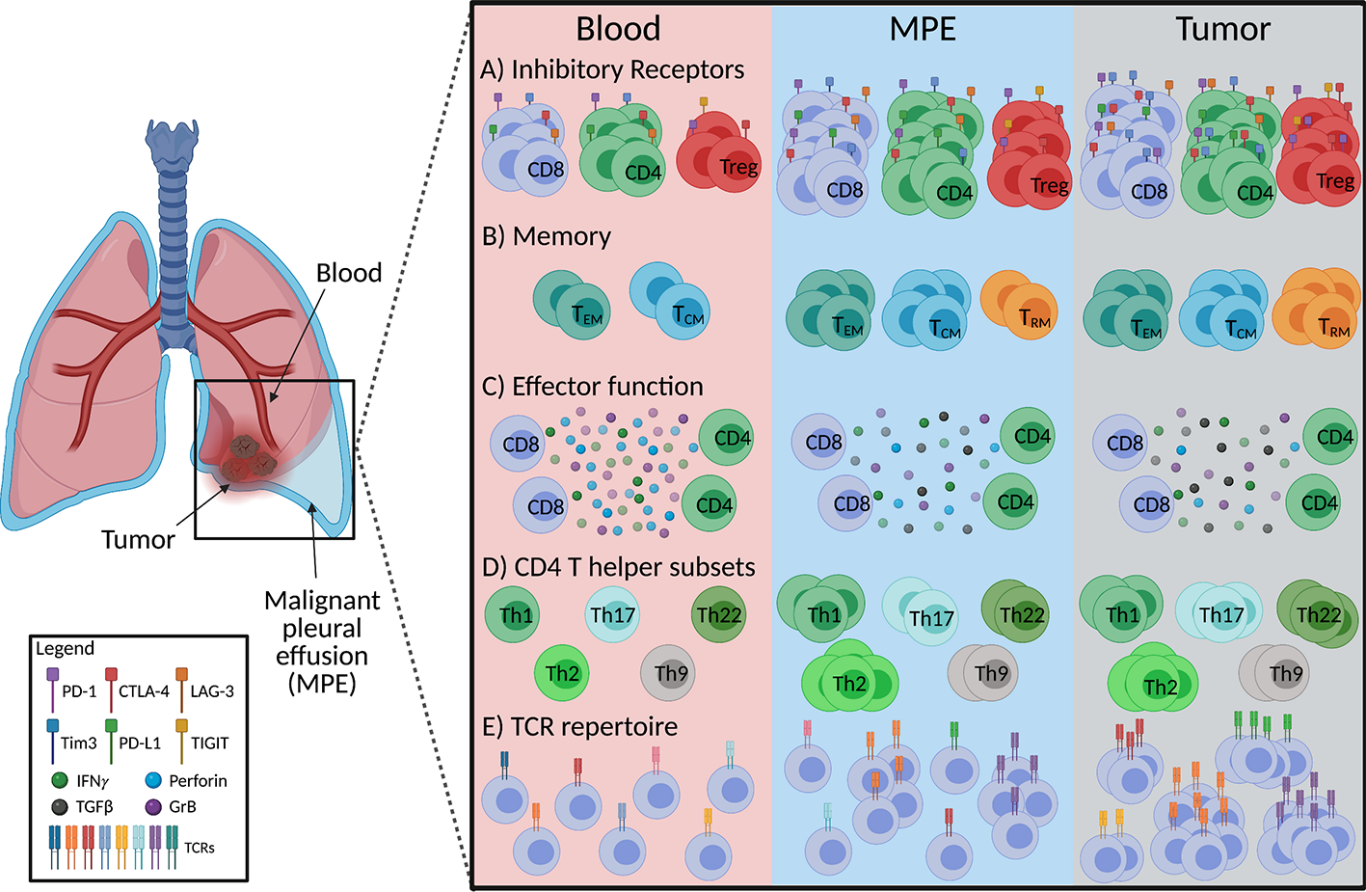
Schematic diagram summarizing characteristics of MPE-derived T cells in comparison to tumor and peripheral blood in mesothelioma and lung cancer. **(A)** Frequencies of CD8^+^, CD4^+^ and CD4^+^ regulatory (Treg) T cells expressing inhibitory receptors in the MPE are similar to tumor infiltrating T cells, however co-expression of inhibitory receptors on T cells is greater in tumors than MPE. **(B)** MPE and tumor contain greater proportions of effector memory (T_EM_) and central memory (T_CM_) T cells than the circulation with tissue resident memory T cell (T_RM_) frequencies the greatest at the tumor site. **(C)** Production of T cell effector cytokines (IFN*γ*, granzyme B; GrB, perforin) are similar between the MPE and tumor infiltrating T cells but lower than those in peripheral blood. **(D)** MPE are likely enriched with a Th2 phenotype along with a greater proportion of Th1, Th2, Th17, Th9, Th22 than circulating T cells, while tumors display a greater frequency of Th17 and Th22 than MPE. Th9 between tumor and MPE is undefined. **(E)** MPE contains tumor reactive T cells, implying a more clonal T cell receptor (TCR) repertoire than the peripheral blood. Figure created with BioRender.com.

The expression of inhibitory checkpoint receptors, enrichment of CD4^+^ regulatory T cells, presence of CD8^+^ memory T cells and impaired cytotoxic, effector T cell function in MPEs suggest that they exist in an immunosuppressed environment. They are more similar to TILs than peripheral blood T cells (**Figure 1**). However, there are reported differences in the CD4^+^/CD8^+^ ratios, co-expression patterns of checkpoint receptors, and CD4^+^ Th subtypes between TILs and MPE T cells. The similarities in antigen-specificity, or TCR usage of T cells between the two compartments are also unknown. Characterizing the phenotypes of T cell clones at both sites would help researchers understand how the MPE or TME shapes the development of these cells. Next, we review how therapies could shape the phenotype of MPE T cells, because such changes could inform biomarker development.

## Changes in MPE-Derived T Cells Following ICB Therapy

Serial analyses of MPE-derived T cells in patients undergoing ICB are rare, but a study of MPE that developed after ICB has been reported. Ikematsu and colleagues characterized T cells in MPE samples drained from lung cancer patients after ICB. There were greater frequencies of CD4^+^TIM-3^+^, CD4^+^TIGIT^+^ and CD8^+^PD-L1^+^ MPE T cells from ICB treated patients compared to MPEs from chemotherapy treated patients (105). However, there were no differences in frequencies of CD8^+^ and CD4^+^ MPE T cells expressing PD-1, TIM-3, TIGIT, PD-L1 or IFN*γ* between responders and non-responders to anti-PD-1 therapy (105, 106). Interestingly, post treatment frequencies of Th17 (CD4^+^IL-17^+^) and CD4^+^LAG-3^+^ T cells in the MPE negatively associated with clinical outcome to anti-PD-1 ICB (105). Two NSCLC patients who became resistant to anti-PD-L1 therapy and developed recurrent MPE had increased frequencies of effector memory (CCR7^-^CD45RA^-^) CD8^+^ T cells and TIM-3 or CTLA-4 expressing CD8^+^ and CD4^+^ T cells cells in the MPE post-treatment, but this was compared to untreated rather than responding patients (107). Together this suggests inhibitory receptor expression increases on MPE T cells in anti-PD-1 and anti-PD-L1 treated patients. There are no studies which have analyzed serial samples of MPE in ICB treated patients.

MPE T cells have been studied in serial samples from patients treated with chemotherapy. Longitudinal analysis of MPE in a mesothelioma patient identified that the percentage of CD3^+^ T cells decreased in the MPE following 4 cycles of cisplatin-pemetrexed based chemotherapy, producing a partial response (10). When on-treatment changes were examined, the first dose of methotrexate chemotherapy reduced total CD3^+^ T cells in the MPE but these frequencies returned back to baseline levels after the second dose of methotrexate in NSCLC patients (108). Frequencies of MPE CD4^+^ T cells increased, regulatory T cells decreased and CD8^+^ T cells were unchanged following methotrexate. In terms of T cell function, it increased frequencies of MPE-derived IFN*γ* ^+^ and IL-2^+^ T cells (108). This suggests that the MPE environment is dynamic, and changes in MPE T cells can be shaped by therapies, similar to T cells in other compartments (65, 66, 109–111).

There are very few studies of serial analyses of MPE-derived T cell phenotype, function and antigen-specificity, and how they associate with ICB outcomes. However, those studies inform us of potential T cell phenotypes from TILs and MPEs that are of interest because they could associate with ICB responses (**Table 1**). Serial analysis of MPE T cells in patients undergoing ICB therapies would be greatly informative.

**Table 1:** Intratumoral T cell characteristics that associate with clinical benefit to ICB in lung cancer patients.

T cell characteristic	Cancer	Pre- or post-treatment	ICB	Ref.	Also found in MPE?
>1% CD8^+^PD-1^hi^ T cells	NSCLC	pre	Nivolumab	(34)	Undefined
Low-PD-1-to-CD8 ratio	NSCLC	pre and post	Nivolumab	(112, 113)	Post-treatment: not reported (105)
High PD-1 transcripts	NSCLC	pre	Nivolumab	(114)	Undefined
CD8^+^PD-1^hi,^ CD4^+^Foxp3^+^PD-L1^hi^	NSCLC	post	Nivolumab	(115)	Undefined
High PD-L1 transcripts	NSCLC	pre	Nivolumab	(114)	Undefined
High CD8:CD3 ratio	NSCLC	pre and post	Nivolumab	(112, 113)	Undefined
>70% TIM-3^+^IL-7R^-^ of CD8^+^CD103^+^ T_RM_	Lung Cancer	pre and post	Nivolumab	(73)	Undefined
High IFNγ mRNA	NSCLC	post	Nivolumab	(79)	Undefined
High activated CD4 T cell signatures with IFN, Th2, IL-17A, IL-26 related genes	NSCLC	pre	Nivolumab	(114)	Post-treatment CD4^+^IL-17^+^ T cells associated with no benefit to ICB (105)
Increased TCR clonality with expanded TCR clones	NSCLC	post	Nivolumab	(38)	Undefined

NSCLC, non-small cell lung cancer.

## Benefits, Opportunities and Challenges for Developing MPE-Derived T Cell Biomarkers

The potential of MPE-derived T cells as a biomarker for therapy responses is attractive for several reasons (**Table 2**). Firstly, MPE-derived T cells may be more closely related to TILs than circulating T cells. The presence of memory CD8^+^ T cells that express inhibitory receptors and CD4^+^ regulatory T cells in MPEs suggest that T cell responses are suppressed, similar to the TME. Furthermore, the MPE environment also consists of tumor cells, MDSCs and immunosuppressive cytokines that may shape T cell phenotype in a similar manner to the TME. Secondly, because pleural fluid is often serially drained, a dynamic biomarker could be developed. We previously argued that not all determinants of ICB response can be found prior to treatment, and changes in TME or blood that occur early on treatment could offer a more accurate, dynamic biomarker of response. Indeed, changes in T cell repertoire phenotype, diversity, and immune gene signatures early during ICB treatment correlate with ICB responses in murine and clinical studies (36, 75, 116–119). While most studies of tumor and blood suggest that changes in CD8^+^ T cells correlate with ICB outcomes, other T cell populations in the MPE, such as CD4^+^ helper T cells, could also be predictive of ICB outcomes. Regular drainage of MPEs provides a unique opportunity to study these dynamic changes. Although this review focuses only on T cells, how MPE-derived T cell frequencies and phenotypes change in relation to other components in the fluid, such as tumor cell, MDSC numbers, and suppressive cytokine levels is informative for biomarker development.

**Table 2:** Benefits and limitations for using the MPE to develop T cell biomarkers for ICB therapy response.

Benefits	Limitations
• MPE-derived T cells are similar in phenotype to tumor infiltrating lymphocytes (TILs) • Ability to develop a dynamic biomarker as multiple fluid drainage due to MPE recurrence is common for thoracic cancer patients • Opportunity to perform high-throughput sequencing technologies i.e. RNAseq and TCRseq on the anti-tumor immune response where tumor biopsies are limited, particularly in malignant mesothelioma	• Fluid volume, cellularity, number and timing of drainage events varies between patients • External factors such as lung diseases, inflammation and infection could alter MPE-derived T cells • Improved MPE treatment regimens that cause pleural space destruction to prevent fluid recurrence.

**Figure 2:**
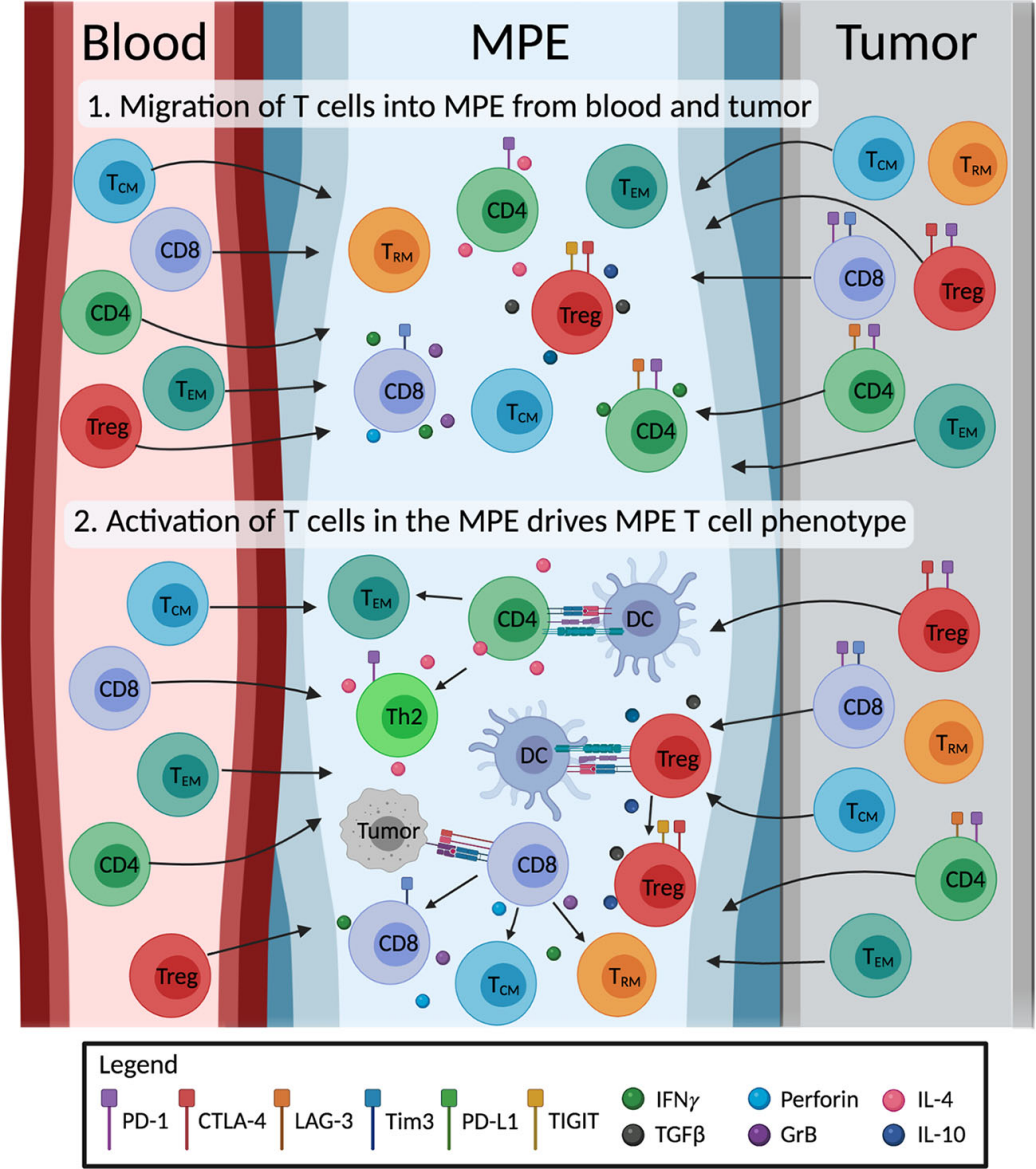
Illustration of the proposed origin and development of MPE T cells. The impact of the MPE environment on T cell differentiation is unclear. We hypothesize that 1) MPE acts as a sink, containing a mix of T cells originally from the blood and the tumor site. 2) MPE environment including cytokines and other cells (e.g. tumor cells, dendritic cells; DC) drive changes in phenotype of MPE T cells. MPE T cells differentiate into effector subtypes, producing immunostimulatory (IFN*γ*, perforin, granzyme B; GrB, IL-4) or immunosuppressive (TGF-β, IL-10) cytokines; exhausted T cells expressing inhibitory receptors; and differentiate into memory T cells (i.e. effector (T_EM_), central (T_CM_) and tissue resident (T_RM_) memory T cells). Figure created with BioRender.com.

MPE-derived T cells exhibit memory phenotypes indicative of chronic antigen-specific activation. The antigen-specificity of MPE T_EM_ and T_RM_ cells, and how they change with therapy are of great interest. It is promising that tumor-reactive T cells can be expanded from the MPE, but the overlap in antigen-specificities between TILs and MPE T cells, if any, are unknown. TCR sequencing offers a complementary method to study the extent of clonal overlap between TILs, blood and MPE-derived T cell populations, and to track the changes in antigen-specific T cells without prior knowledge of any tumor antigens. The extent in which the MPE environment drives T cell differentiation is unclear. We speculate that MPE consist of T cells that have migrated from the local tumor and blood. However, to what extent the MPE environment changes T cell phenotype is unclear. It is possible that further activation and differentiation of T cells in the MPE drives the distinct phenotypes of MPE-derived T cells (**Figure 2**). Single cell technology is a powerful tool to comprehensively study the interactions of different cells in the MPE, and will greatly help our understanding in this area. The transcriptome and TCRαβ usage of individual T cells can be determined, allowing researchers to match phenotypes to individual T cell clones. These TCRs of interest can be subsequently screened for tumor reactivity. Single cell technology is now used in numerous studies of TMEs, and can be applied to MPE samples.

However, there are some limitations with studying MPEs (**Table 2**). The volume of fluid drained, and cellularity of MPE samples varies between patients (62, 120, 121). In some instances, MPE cell numbers are too few for meaningful downstream analysis, especially for rare T cell subsets. Even though longitudinal analysis can be performed with MPE samples, the number of drainage events vary between patients and the timing of them cannot be predicted. Furthermore, differences in MPE immunophenotype measured over time are not always attributed to tumor or treatment. External factors such as infections and lung inflammation could alter the T cell phenotype and are confounding factors that have to be accounted for (122–124). Lastly, management regimens to treat MPEs including talc pleurodesis and VATS pleurodesis and pleurectomy, which is a palliative therapeutic option for malignant pleural mesothelioma patients, aim to obliterate the pleural space and prevent MPE recurrence. This then eliminates the opportunity to serially sample the MPE for biomarkers of response to therapy.

## Conclusions and Future Directions

Although the cellular components of MPE have been studied extensively, recent developments in cancer immunotherapy and the need for biomarkers of response have led researchers to focus on MPE T cells. These cells share phenotypic features with TILs, but further study is required to elucidate if MPE T cells are truly reflective of their tumor counterparts. We think that dynamic analyses of MPE T cells in relation to ICB outcomes will lead to a robust and clinically useful ICB response biomarker.

## Author Contributions

NP wrote the article and made the figures. JK, WL, RL, AN, AM, and JC critically revised the manuscript. All authors contributed to the article and approved the submitted version.

## Funding

NP was supported by Cancer Council WA and UWA Richard Walter Gibbon Medical Research scholarships. JK was supported by icare Dust Diseases Board. WL was supported by a Simon Lee Fellowship, an NHMRC Fellowship, and a Cancer Council WA fellowship. AM was supported by grants and fellowship from Cancer Council WA, Raine Medical Research Foundation and icare Dust Diseases Board. JC was supported by grants and fellowship from the Raine Medical Research Foundation, Cancer Council WA, WA Department of Health, and icare Dust Diseases Board. The National Centre for Asbestos Related Diseases receives funding through the National Health and Medical Research Council Centres of Research Excellence scheme APP1197652.

## Conflict of Interest

The authors declare that the research was conducted in the absence of any commercial or financial relationships that could be construed as a potential conflict of interest.
